# Interventional Radiology Management of Renal Artery Stenosis After Kidney Transplant: Single-Center Experience and Management Strategies

**DOI:** 10.3390/diagnostics15131592

**Published:** 2025-06-23

**Authors:** Ahmad Mirza, Munazza Khan, Usman Baig, Shameem Beigh, Imran Gani

**Affiliations:** 1 Surgery Division, Wellstar MCG Health, Augusta University, Augusta, GA 30912, USA; 2 Medical University-Pleven, 1, Saint Kliment Ohridski Street, 5800 Pleven, Bulgaria; 3 Department of Nephrology, Hypertension and Transplant Medicine, Wellstar MCG Health, Augusta University, Augusta, GA 30912, USA

**Keywords:** interventional, radiology, stenosis, renal, transplant, allograft, hypertension

## Abstract

**Background/Objectives:** The course of treatment for renal artery stenosis following renal transplantation depends on the severity of the condition. Mild cases are typically managed medically, while more significant stenosis with flow limitation and graft dysfunction requires percutaneous intervention. Surgical treatment is generally reserved as a last resort. This study aimed to evaluate the outcomes of interventional radiology in managing renal artery stenosis at our transplant center. **Methods:** The electronic medical records of patients who underwent renal transplantation at our center between January 2020 and December 2024 were reviewed to identify cases of renal artery stenosis and their subsequent management through interventional radiology. Sociodemographic and clinical data were collected for both recipients and donors. Data analysis was performed using SPSS version 26. **Results:** Out of the total 368 patients who received renal allograft at our center from January 2020 to December 2024, 25 patients were confirmed with duplex ultrasound to have renal artery stenosis. The majority of affected patients were African American, had Class I Obesity and presented with cardiovascular co-morbidities. The mean time from transplant to the diagnosis of RAS was 4.25 (SD ± 3.81) months. The mean serum creatinine level at presentation was 2.54 (SD ± 1.21 mg/dL). All 25 patients underwent digital subtraction angiography, and 24 patients were confirmed to have renal artery stenosis requiring further intervention. The creatinine levels at one week, three months and one year post-intervention were 2.12 (SD ± 1.00), 1.83 (SD ± 0.63) and 2.15 (SD ± 1.68) mg/dL, respectively. **Conclusions:** Percutaneous interventional treatment for renal artery stenosis is associated with improvements in hemodynamic parameters and the stabilization of allograft function. Follow-up is needed to monitor for the potential occurrence of restenosis.

## 1. Introduction

Renal artery stenosis (RAS) is a well-known complication of kidney transplant (KT) surgery, with its incidence ranging from 1% to 23% in KT recipients [1]. It most often affects the arterial anastomosis, with a majority of cases presenting within 6 months of the procedure [2]. As the most common vascular complication of KT surgery and its impacts on allograft functioning, the development of RAS must be promptly identified and addressed to prevent graft deterioration and loss. RAS represents almost 75% of KT vascular complications, although its prevalence may increase due to the augmented criteria for potential KT donors and recipients, which now incorporate an older population [3].

Many of the risk factors associated with RAS are modifiable, including recipient comorbidities such as hypertension (HTN) and diabetes mellitus (DM), as well as peri-operative cold ischemia time and kidney donation from deceased donors. Other risk factors, such as delayed graft function (DGF) and acute graft rejection (AGR), have also been associated with RAS [4]. RAS causes the hypoperfusion of the kidney allograft and may activate the renin–angiotensin–aldosterone system (RAAS), resulting in hypertension that is often refractory to medical treatment. This can lead to fluid overload, which causes the increased morbidity and mortality seen in KT recipients with RAS [5]. RAS cases are commonly diagnosed with the clinical application of duplex ultrasound (US) and magnetic resonance angiography (MRA) [1]. Prevention, early detection and timely treatment are therefore key strategies in reducing the morbidity and mortality associated with RAS in kidney transplant recipients.

We aimed to evaluate the outcomes of the percutaneous radiological intervention in transplant artery renal stenosis, including the need for re-intervention, and to assess its impact on renal function post-procedure. The findings of this study are intended to contribute to a better understanding of the efficacy and clinical value of interventional radiology in the context of RAS and its impact on improving the overall survival of the kidney allograft.

## 2. Methods

Ethical approval for the study was obtained from the institutional review board 2294849-1. Electronic medical records from January 2020 to December 2024 were reviewed to identify patients who underwent renal transplantation. A total of 368 transplant recipients were included. Two independent researchers analyzed the data to identify cases of RAS. Sociodemographic and clinical information for both recipients and donors was collected and verified for accuracy. Clinical data at the time of presentation were collected, including initial radiological imaging, which was reviewed to determine the location of the stenosis. The time duration from transplant to initial diagnosis and subsequent intervention was calculated. Information on repeat interventions performed by the interventional radiology department was collected, along with follow-up data for all patients.

All patients received a kidney allograft from the deceased donation registry allocated by United Network of Organ Sharing (UNOS, Richmond, VA, USA). All patients were transplanted with an aortic Carrel patch to the recipient external iliac artery. All patients received standard pre-operative and post-operative care.

The data were analyzed using the Statistical Package for the Social Sciences (SPSS), version 26 (Chicago, IL, USA). All recipient and donor parameters were stratified and analyzed accordingly, with the results presented in tabulated format.

## 3. Results

In total, 25 patients were identified who had a duplex-confirmed diagnosis of the renal artery stenosis of the transplanted kidney. The mean patient age was 35.74 ± 17.25 years. The Kidney Donor Profile Index (KDPI) for donor kidneys ranged from 35% to 85%. Most patients had class I obesity and cardiovascular comorbidities. The baseline clinical characteristics of both donors and recipients are summarized in Table 1. All patients had a fully established graft function and were dialysis-independent before the diagnosis of RAS.

The mean time from transplant to the diagnosis of RAS was 4.25 ± 3.81 months. At the time of diagnosis, the mean systolic blood pressure was 152.56 ± 22.91 mmHg, and diastolic blood pressure was 78.26 ± 16.65 mmHg. The average serum creatinine level was 2.54 ± 1.21 mg/dL. Details of these parameters including their mean, median, mode, standard deviation, standard error and percentiles are detailed in Table 2.

The most common site of stenosis was at the anastomosis (45.8%), followed by the proximal part of the transplanted renal artery (33%). Among the 24 patients who underwent initial angioplasty, 5 patients required reintervention. The details of the cases are presented in Table 3. There were no reported complications from angioplasty intervention both in patients who required angioplasty earlier (within 3 months of transplant) versus later interventions.

The follow-up data show the greatest reduction in mean systolic pressure at 1 week, with a value of 137.57 ± 20.39 mmHg. The lowest reduction was at the one year interval, with a value of 143.46 ± 17.46 mmHg. The lowest mean serum creatinine level was recorded three months after the intervention at 1.83 ± 0.63 mg/dL. The average number of antihypertensive medications taken by the subjects remained three throughout the first year following the intervention. Detailed information on recipient characteristics at 1 week, 3 months and 12 months after the intervention is presented in Table 4.

## 4. Discussion

T-RAS is a significant cause of graft loss and death in KT patients. However, these complications can be reduced through effective treatment. It is important to recognize early clinical signs and prompt imaging to diagnose RAS [6]. The diagnosis of T-RAS can be conclusively established through invasive angiography, although this method of diagnosis increases the risk of complications such as contrast nephropathy, hematoma, pseudoaneurysm and thromboembolism [5]. Less invasive techniques include computed tomographic angiography (CTA), magnetic resonance angiogram (MRA) and duplex ultrasonography (US). The sensitivity and specificity of CTA in diagnosing T-RAS are similar to those of invasive angiography. Though CTA is less invasive, it is associated with the risk of inducing nephropathy through the use of iodinated contrast. The sensitivity of MRA in diagnosing T-RAS is over 90%. It also does not expose the patient to the harmful effects of contrast dye or ionizing radiation [7]. However, it is both more expensive and less accessible than CTA, and it is not recommended to patients with previous kidney stents due to the potential for metal artifacts that lead to inaccurate projections of images. Finally, Doppler US is widely available and cost-effective in detecting T-RAS. Its main limitation is its dependence on the technician’s proficiency. Factors such as an obese patient and overlying bowel gas may also affect its diagnostic capacity [1,5,7]. In our study, we initially evaluated our patients with duplex US, and the diagnosis of T-RAS was then confirmed and managed by interventional radiology imaging.

Once the diagnosis of T-RAS has been established, both medical and interventional treatment options can be utilized in KT recipients. Medical treatment with antihypertensives may suffice for patients with minimal stenosis. All patients in our series were treated initially with antihypertensive medications and close monitoring before proceeding for angiographic intervention. Patients with significant stenosis and allograft dysfunction will require interventional techniques [8]. Lifestyle modifications, antihypertensives, antiplatelet drugs and lipid-lowering agents are recommended in both native and transplant T-RAS patients [9]. If HTN is unresponsive to medical management or if a decline in graft function determined through an increase in creatinine is observed, then percutaneous or surgical interventions are recommended [10]. Percutaneous transluminal angioplasty (PTA) +/− stenting is considered as a first-line treatment for medically refractory T-RAS, although surgical treatment has been shown to be efficacious in patients for whom this procedure is unsuitable. A study by Rouer et al. on surgically treated T-RAS in transplanted kidneys described a high success rate, with adequately controlled blood pressure and no graft loss 5 years post-operatively in all patients [11]. Indications for surgical treatments include failed PTA attempt(s) or the futility of PTA due to the location of the stenosis [10].

In patients where PTA is feasible, its high procedural success rate and patency duration make it the endovascular procedure of choice [11]. Chew et al.’s 10-year and Greenstein et al.’s 6-year retrospective studies on PTA used to treat T-RAS in KT patients found high levels of clinical success (76.9%) and improvement in graft functioning (76%), respectively [12,13]. However, no evidence related to anastomosis site leak or rupture related to angioplasty was documented [12,13]. Angioplasty is considered a safe option for the management of T-RAS even < 3 months since transplant as demonstrated in our patient population. A statistically significant reduction in antihypertensive use and improvement in kidney allograft function was seen post-procedurally, making PTA an effective treatment option in medication-resistant T-RAS [14,15]. The 10-year graft survival and patient survival rates were shown to be similar in PTA-treated KT patients when compared to a matched control group, indicating the long-term success of managing T-RAS patients with PTA [16]. However, in a cohort of KT patients without medically refractory T-RAS, the superiority of PTA over medical management has not been unequivocally established. Seratnahaei et al. compared studies showing improvement in clinical parameters in PTA-treated patients with studies that showed no advantages of PTA [10]. While a majority of the studies detailed have demonstrated the benefit of PTA, some studies, including Seratnahaei et al.’s, did not show any benefit [10]. Nevertheless, it must be noted that in the studies that do not show the superiority of PTA over conservative management in KT T-RAS patients, the inferiority of the procedure is also not established [10]. Larger-scale randomized control trials are necessary to decisively determine whether medical treatment or PTA is more effective in treating T-RAS in KT patients who are responsive to medical management.

In our study, all 24 of our post-KT RAS patients were treated with PTA, of which 15 (63%) patients also had stents placed. Stent placement is indicated when >25% of the stenosis of the vessel remains after dilation with PTA [17]. We followed the same guidance in our cohort of patients. Differences in studies exist regarding whether PTA and stenting shows improved outcomes in KT RAS patients versus PTA alone. A systematic review of 54 articles with 1522 KT patients has supported the use of adjuvant stents in PTA, as the stenting group showed a significant decrease in serum creatinine levels, indicating improved graft function [18]. In patients with recurrent stenosis, stenting has been shown to slow down or halt re-stenosis, control patient blood pressure, lower serum creatinine levels and reduce the number of antihypertensives administered per patient [19,20]. However, there are also individual studies that find no noteworthy difference between stenting groups and PTA-only groups as regards reductions in creatinine and MAP [21,22]. When taking into account complications from PTA +/− stenting, the overall complication rate is approximately 10%, with the most common complications being arterial dissection, pseudoaneurysms and hematomas [2,18].

PTA stenting options constitute both balloon-expandable stents (BESs) and self-expanding stents (SESs) for use in T-RAS. There is no clear indication regarding the use of one stent over the other. Both stents have high technical success rates of over 95%, each with its own pros and cons. The BESs, which are inflatable, are both advantageous and potentially injurious to the vessel, whereas SES can be deployed with low pressure [23,24]. A systematic review of 20 studies by Qureshi et al. comparing BES and SES use in intracranial arterial stenosis found lower 30-day stroke and mortality rates with BESs [25]. Conversely, Krankenburg et al. described their experience of 660 patients with iliac artery occlusive disease. Patients treated with an SES were shown to have reduced 12-month recurrent stenosis and a significantly decreased need for target lesion revascularization [26]. Studies comparing the outcomes with BESs and SESs in T-RAS in particular are currently lacking.

Choices regarding stents also vary with regard to the use of a medication coating, with the option of placing either bare metal stents (BMSs) or drug-eluting stents (DESs) in T-RAS. Due to their technical placements being identical, BMSs and DESs have similar clinical success and adverse effect rates [27]. PTA with either stent type has been proven to be an effective short- and long-term treatment option in lowering serum creatinine and blood pressure in RAS KT recipients [28,29]. In atherosclerotic T-RAS, patients treated with a DES displayed relatively greater decreases in blood pressure and the use of antihypertensives than the BMS group, which showed a higher incidence of decline in kidney function [30]. Extrarenal studies comparing the use of BMSs vs. DESs found that DESs yield superior results. Longer stent patency and a reduced risk of in-stent restenosis have been found with the use of DESs in transplant hepatic artery stenosis and intracranial arterial stenosis, respectively [31,32,33].

DESs are coated with antiproliferative medication to minimize restenosis, with the first DES approved by the FDA utilizing sirolimus to prevent vascular smooth muscle migration and proliferation [34]. More recently, heparin-coated DESs have been introduced. When quantified in vitro using immunofluorescence and electron microscopy, heparin-coated stents (HCSs) sustain less fibrin and platelet accumulation than BMSs due to their lower platelet and immunologic activation [35]. In vivo, in a study using porcine models and in a study on patients with coronary artery disease, PTA alone or PTA with BMS were found to initiate a greater immune response, larger areas of restenosis and lower adverse effect-free survival than that with HCS [36,37]. The rare complication of stent thrombosis (ST) is most commonly seen with first-generation DESs that contain sirolimus or paclitaxel, necessitating the use of extensive dual antiplatelet therapy (DAT) [38]. Despite being relatively uncommon, ST can lead to significant morbidity and mortality [39]. Other complications for which DAT is warranted post-stenting include transient ischemic attacks (TIAs) and stroke. The risks of complications such as ST, major bleeding and cardiovascular events are not statistically different between the use of DAT and single antiplatelet therapy (SAT), thus providing no clinical basis for revising DAT to SAT in patients undergoing stent placement [40,41]. A study by Valgimigli et al. has demonstrated no additional benefit of a longer duration of DAT over a shorter duration (3 months versus 1 month) as regards MI or all-cause mortality, with shorter durations of DAT conferring lower bleeding risk [42]. Future advancements in stent design and the development of second-generation stents reduce the risks of intimal damage and inflammation, which potentially modulate the requirement for protracted DAT and subsequently mitigate the risk of bleeding [38].

Once T-RAS in KT recipients has been diagnosed and treated, routine follow-up is necessary for continuing to monitor kidney graft function. We performed surveillance duplex imaging of the kidney allograft every three months up to one year from the date of angioplasty, We perform 6 monthly duplex imaging of the kidney allograft every 6 months after Although T-RAS tends to stabilize over time, many patients may experience a relapse of the stenosis. The preferred treatment of choice for post-KT RAS is PTA +/− stenting, and the 1-year patency of which ranges from 72% to 94% [2]. Recurrent stenosis can be monitored using serum creatinine and blood pressure measurements, as well as regular Doppler US evaluations. If Doppler US assessment shows re-stenosis, digital subtraction angiography can be repeated [43].

## 5. Conclusions

Once diagnosed, the treatment of T-RAS through medical, percutaneous or surgical means is indicated based on the severity of the stenosis and the need to escalate management. Our study shows a single-center experience in managing T-RAS, but the study is limited by the number of cases presented. PTA +/- stenting is the first-line treatment for T-RAS, with different options available depending on the stent type and deployment. There was a stabilization in renal function and improvement in hemodynamic parameters following the intervention. Regular follow-up post-T-RAS is recommended to continue monitoring graft function and evaluate the risk of recurrent stenosis.

## Figures and Tables

**Table 1:** Clinical characteristics of renal allograft recipients and donors.

			**Frequency (*n*)**	**Percentage (%)**
***Baseline Donor Characteristics***				
**Mean age ± SD** (years)			35.74 ± 17.25	-
**KDPI Score Classification**	<35%		10	40.0
	35–85%		14	56.0
	>85%		1	4.0
**Mean Cold Ischemia Time (minutes)**			1424 ± 455.34	-
***Baseline Recipient characteristics***				
**Mean age ± SD** (years)			53.29 ± 12.71	-
**BMI (Kg/m^2^)**	**Normal**	18.5–24.9	5	20.0
	**Overweight**	25–29.9	6	24.0
	**Class I Obesity**	30–34.9	9	36.0
	**Class II Obesity**	35–39.9	5	20.0
**Ethnicity**	**African American**		20	80.0
	**Caucasian**		4	16.0
	**Hispanic**		1	4.0
**Co-morbidities**	**Cardiovascular Disease**		22	88.0
	**Diabetes Mellitus**		12	48.0
	**Hyperlipidemia**		9	36.0
	**Nephritis**		2	8.0
	**History of Prior Surgery**		3	12.0

**Table 2:** Clinical profile of recipient at time of renal artery stenosis detection. RAS: renal artery stenosis. SBP: systolic blood pressure. DBP: diastolic blood pressure. SCr: serum creatinine; SD: Standard Deviation; SE: Standard Error.

		Mean	SD	SE
**Elapsed Time Post-Transplant Until RAS Diagnosis**	**Days**	148.25	116.77	23.83
	**Months**	4.25	3.81	0.77
**SBP (mmHg)**		152.56	22.91	4.77
**DBP (mmHg)**		78.26	16.65	3.47
**SCr (mg/dL)**		2.54	1.21	0.25
**Prescribed Antihypertensive Medications**		3.0	1.41	0.29

**Table 3:** Location of stenosis and interventional radiology management in cases of renal artery stenosis.

Case No.	Location of Stenosis	Initial Intervention	IR Re-Intervention in 12 Months
1.	Anastomosis	Balloon expandable—6 mm × 18 mm	-
2.	Anastomosis	Balloon expandable—6 mm × 20 mm + Angioplasty	-
3.	Mid	Angioplasty	-
4	Anastomosis	Angioplasty	Angioplasty and stent
5	Anastomosis	Balloon expandable—5 mm × 5 mm + Angioplasty	Angioplasty of stent
6.	Proximal	Balloon expandable—6 mm × 18 mm + Angioplasty	-
7.	Anastomosis	Bare metal—6 mm × 19 mm + Angioplasty	-
8.	Proximal	Angioplasty	-
9.	Proximal	Balloon expandable—4 mm × 6 mm + Angioplasty	-
10.	Proximal	Balloon expandable—5 mm × 15 mm + Angioplasty	-
11.	Proximal	Angioplasty	-
12.	Anastomosis	Angioplasty	-
13.	Anastomosis	Angioplasty	-
14.	Mid	Angioplasty	Stent (4 mm × 19 mm)Angioplasty of stent
15.	Anastomosis	Balloon expandable—6 mm × 2 mm + Angioplasty	-
16.	Proximal	Balloon expandable—5 mm × 15 mm	Angioplasty of stent
17.	Anastomosis	Balloon expandable—6 mm × 18 mm	-
18.	Hilum	Angioplasty	Stent Angioplasty of stent
19.	Mid	Balloon expandable—2.5 mm × 15 mm and 3.0 mm × 15 mm + Angioplasty	-
20.	Anastomosis	Balloon expandable—5 mm × 15 mm	-
21.	Anastomosis	Balloon expandable—6 mm × 18 mm	-
22.	Proximal	Angioplasty	-
23.	Proximal	Balloon expandable—4 mm + Angioplasty	-
24.	Hilum	Balloon expandable—5 mm × 40 mm + Angioplasty	-
25.	Proximal	Angiogram only	-

**Table 4:** Recipient characteristics after intervention at 24 h, 1 week and 3 and 12 months follow-up. SBP: systolic blood pressure; DBP: diastolic blood pressure; SCr: serum creatinine. Anti-HTN: antihypertensive; SD: standard deviation; SE: standard error.

	Post-Procedure (24 h)			1 Week			3 Months			12 Months		
	Mean	SD	SE	Mean	SD	SE	Mean	SD	SE	Mean	SD	SE
**SBP (mmHg)**	147	17.89	3.98	137.57	20.39	4.45	140.63	16.27	3.73	143.46	17.46	4.51
**DBP (mmHg)**	81	12.01	2.9	75.00	14.00	2.8	72.9474	13.04	2.99	76.40	14.19	3.66
**SCr (mg/dL)**	2.44	1.23	0.24	2.12	1.00	0.21	1.83	0.63	0.14	2.15	1.68	0.43
**Anti-HTN**	3.0	1.41	0.29	3.0	1.40	0.28	2	1.32	0.31	2	1.36	0.33

## Data Availability

The data that support the findings of this study are available on request from the corresponding author. The data are not publicly available due to privacy or ethical restrictions.
